# Impact of the RTS,S Malaria Vaccine Candidate on Naturally Acquired Antibody Responses to Multiple Asexual Blood Stage Antigens

**DOI:** 10.1371/journal.pone.0025779

**Published:** 2011-10-12

**Authors:** Joseph J. Campo, Carlota Dobaño, Jahit Sacarlal, Caterina Guinovart, Alfredo Mayor, Evelina Angov, Sheetij Dutta, Chetan Chitnis, Eusebio Macete, John J. Aponte, Pedro L. Alonso

**Affiliations:** 1 Centre de Recerca en Salut Internacional de Barcelona, Hospital Clinic / Universitat de Barcelona, Barcelona, Spain; 2 Centro de Investigação em Saúde de Manhiça, Manhiça, Mozambique; 3 United States Military Malaria Vaccine Program, Walter Reed Army Institute of Research, Silver Spring, Maryland, United States of America; 4 International Centre for Genetic Engineering and Biotechnology, New Delhi, India; Museum National d'Histoire Naturelle, France

## Abstract

**Background:**

Partial protective efficacy lasting up to 43 months after vaccination with the RTS,S malaria vaccine has been reported in one cohort (C1) of a Phase IIb trial in Mozambique, but waning efficacy was observed in a smaller contemporaneous cohort (C2). We hypothesized that low dose exposure to asexual stage parasites resulting from partial pre-erythrocytic protection afforded by RTS,S may contribute to long-term vaccine efficacy to clinical disease, which was not observed in C2 due to intense active detection of infection and treatment.

**Methodology/Principal Findings:**

Serum collected 6 months post-vaccination was screened for antibodies to asexual blood stage antigens AMA-1, MSP-1_42_, EBA-175, DBL-α and variant surface antigens of the R29 laboratory strain (VSA_R29_). Effect of IgG on the prospective hazard of clinical malaria was estimated. No difference was observed in antibody levels between RTS,S and control vaccine when all children aged 1–4 years at enrollment in both C1 and C2 were analyzed together, and no effects were observed between cohort and vaccine group. RTS,S-vaccinated children <2 years of age at enrollment had lower levels of IgG for AMA-1 and MSP-1_42_ (p<0.01, all antigens), while no differences were observed in children ≥2 years. Lower risk of clinical malaria was associated with high IgG to EBA-175 and VSA_R29_ in C2 only (Hazard Ratio [HR]: 0.76, 95% CI 0.66–0.88; HR: 0.75, 95% CI 0.62–0.92, respectively).

**Conclusions:**

Vaccination with RTS,S modestly reduces anti-AMA-1 and anti-MSP-1 antibodies in very young children. However, for antigens associated with lower risk of clinical malaria, there were no vaccine group or cohort-specific effects, and age did not influence antibody levels between treatment groups for these antigens. The antigens tested do not explain the difference in protective efficacy in C1 and C2. Other less-characterized antigens or VSA may be important to protection.

**Trial Registration:**

ClinicalTrials.gov NCT00197041

## Introduction

GlaxoSmithKline Biologicals' adjuvanted RTS,S malaria vaccine candidate has repeatedly demonstrated protective efficacy in clinical trials in Africa [1]. It is composed of the NANP central repeat and C-terminal T-cell multi-epitope of *Plasmodium falciparum* circumsporozoite protein (CSP), fused with the S-antigen of hepatitis B virus and combined with an AS adjuvant systems [2], either AS02 (QS21, MPL and an oil-in-water emulsion) or AS01 (QS21, MPL and liposomes) [3]. The RTS,S/AS01 formulation is being evaluated in a Phase III efficacy trial.

The generation of high titer anti-CSP antibodies has been extensively documented following RTS,S vaccination of malaria-naïve adult volunteers [4]. Although certain antibody thresholds have been proposed that may be necessary to achieve protection [5], to date, there is no strict anti-CSP IgG correlate of protection derived from studies involving laboratory-based challenge of vaccinated volunteers with the bite of an infectious mosquito. Additionally, an association has been shown between CSP-specific CD4+ T cell responses and protection in a laboratory challenge model [5]. However, information is lacking on immunological correlates of protection in the face of natural exposure to malaria, which the challenge model cannot provide. Similar efforts in African field trials of RTS,S have confirmed the consistent, high titer generation of CSP-specific antibodies, while cell-mediated immune (CMI) responses have not yet been systematically studied [6]. Interestingly, in field studies where efficacy against infection is the primary endpoint, CSP antibodies seemed to correlated with protection [7], [8], whereas no such correlation could be found with protection against clinical manifestation of disease [7], [9], except in a recent trial of RTS,S/AS02 in infants [10] and a recent analysis in children vaccinated with RTS,S/AS01 where anti-CSP antibody titers 6.5 months after vaccination seemed to correlate with protection [11].

Protection has been observed for up to 43 months following vaccination despite declining levels of CSP-specific antibodies [12], [13], and efficacy measurements remained remarkably stable. This long-term protection observed in Mozambican children differs markedly from the waning protection observed in earlier studies of RTS,S/AS02 in Gambian men and U.S. non-immune adults in the U.S. [14], [15]. This unexpected finding suggests that, in addition to anti-CSP antibodies, other factors may contribute to sustained protection, such as anti-CSP CMI, fine specificity and functionality of CSP-specific antibodies, or acquisition of blood stage immunity greater than that which would be acquired naturally [4]. Indeed, a hypothesis that RTS,S vaccination may affect blood stage immunity was proposed at earlier stages of this vaccine's development [16]. The concept of enhancing naturally acquired immunity through interventions that reduce the blood stage parasite burden was proposed to explain long-term efficacy of intermittent preventive treatment in infants [17]. In the case of RTS,S vaccination, it was hypothesized that low dose parasitemia as a result of partial pre-erythrocytic protection may allow for a more effective immune response to asexual blood stage parasites [18], [19]. The alternative to this hypothesis would be that RTS,S vaccination reduces naturally acquired immune responses to blood stage parasites through reduction in high level exposure.

In the Phase IIb trial in children 1–4 years of age performed in Manhiça, Mozambique, the study was divided into two cohorts, each with different follow up methods and schedules [7]. Cohort 1 (C1) was followed for 43 months post-vaccination by passive case detection (PCD) of clinical malaria, and an efficacy of 30.5% against first or only episode of clinical malaria was observed over the entire follow-up period [13]. The estimated entomological inoculation rate in Manhiça District in 2002 was 38 infective bites per person per year [20]. Cohort 2 (C2) was followed for 6 months post-vaccination by active detection of infection (ADI) by fortnightly and monthly household visits and thereafter by PCD; vaccine efficacy was initially 45.0% against first or only episode of infection, but waning efficacy was observed by 6 months post-vaccination. There were no EIR estimates in this area at the time of the trial, but transmission intensity in C2 was notably higher, as deduced by elevated blood stage immunofluorescence antibody test (IFAT) responses at baseline [7]. In addition to transmission intensity, another key difference was prompt clearing of parasitemia during ADI visits in C2, regardless of parasite density or presence of fever.

Here, we examined the antibody immune responses to multiple asexual blood stage antigens at 6 months post-vaccination, the time when waning efficacy is observed in C2 but not in C1. We hypothesized that in C1, antibodies to asexual blood stage antigens in the RTS,S/AS02 group would be higher than those in the control group. Furthermore, we hypothesized that the magnitude of the difference between C2 vaccine groups would be less than that observed in C1. We measured antibodies to leading blood stage vaccine-candidate antigens AMA-1 [21], MSP-1_42_ [22], [23] and EBA-175 [24], as well as variant surface antigens of the R29 *P. falciparum* culture line (VSA_R29_) and recombinant DBL-α. The R29 culture line exhibits high levels of rosetting, and DBL-α is encoded within *var-*1 of R29 [25]. Each of these antigens has been targeted for vaccine development or for further investigation due to their importance in the blood stage parasite lifecycle, such as merozoite invasion, immune evasion and cytoadherence [25]–[27]. Although it is unknown if there is a causal relationship between antibodies to these antigens and protective immunity, some of these antibodies have shown association with protection in seroepidemiological studies [28]–[30].

## Methods

### Ethics Statement

The study was approved by the Mozambican National Health and Bioethics Committee, the Hospital Clínic of Barcelona Ethics Committee and the PATH Research Ethics Committee, and written informed consent was gathered from parents/guardians.

### Study Design

The samples assessed were obtained as part of a Phase IIb proof-of-concept randomized, controlled trial of the RTS,S/AS02 vaccine administered to 1–4 year-old Mozambican children (ClinicalTrials.gov registry number NCT00197041). The trial design has been described in detail in multiple primary research articles [7], [31], [13], [19].

Serum samples were obtained from cross-sectional blood collections at the start of the single-blind phase of follow-up, corresponding to 6 months after third dose [31], [19]. This timepoint was selected, because it provides a period of 6 months of natural exposure following vaccination and coincides with the beginning of the follow-up period where waning efficacy was observed in C2 [19]. Samples were selected to have an equal representation of C1 and C2 and of older and younger children. To do this, samples were stratified by cohort and by two age groups (≥ or <2 years of age) to create 4 subgroups of the full sample set. Samples were randomly selected in each subgroup. Baseline characteristics of the samples selected, including age group, previous episodes and pre-vaccination blood stage antibody titers, are given in Table S1.

### Recombinant Proteins

Apical membrane antigen 1 (AMA-1) 3D7 strain [21], PfF2 (fragment II of region II of the 175 kDa erythrocyte binding antigen, EBA-175) [32], and the Duffy binding-like alpha (DBL-α) domain of PfEMP-1 [25] were produced at ICGEB. FMP009 (FVO strain of AMA-1), FMP1 and FMP010 (3D7 and FVO strains of MSP-1_42_, respectively) [22], [23], all were produced at WRAIR.

### Suspension array technology

#### Microsphere coupling

A multiplex suspension array technology (SAT) panel was constructed to quantify IgG responses to *P. falciparum* antigens using Luminex xMAP™ (Luminex Corp., Austin, Texas) and the Bio-Plex 100 platform (Bio-Rad, Hercules, CA). xMAP™ beads (regions: 4, 10, 15, 34, 40, and 45) were selected for each antigen. Uncoupled polystyrene 5.6 µm COOH-microspheres (Bio-Rad) were coupled to antigen in 200 µL coupling reactions (2.5×10^6^ microspheres). First, microspheres were washed twice with 100 µL of Wash Solution from the Bio-Rad carboxylated microsphere coupling kit. Microspheres were resuspended by sonication and vortexing and activated using Bead Activation buffer. Sulfo-NHS (N-hydroxysulfosuccinimide) and EDC (1-Ethyl-3-[3-dimethylaminopropyl]carbodiimide hydrochloride) (Pierce, Thermo Fisher Scientific Inc., Rockford, IL) were simultaneously added to reaction tubes at 5 mg/mL each in Bead Activation buffer, and reaction tubes were incubated at room temperature with gentle agitation, protected from light for 20 min. Microspheres were washed with 100 µL PBS, sonicated and vortexed. One microgram of corresponding recombinant protein per million microspheres was added to each reaction tube. A prior titration of the antigen concentrations confirmed that as little as 1 µg of each antigen per million microspheres could be used without changing the saturation levels of the microspheres when assayed with hyperimmune plasma (HIP). Reaction tubes were left at 4° C on a shaker overnight, protected from light. Microspheres were then blocked with 250 µL of 1% bovine serum albumin (BSA) in PBS for 30 min on a shaker at room temperature, protected from light, then centrifuged and washed with 500 µL assay buffer (1% BSA/0.05% sodium azide in PBS, filtered). Coupled microspheres were quantified on a Guava PCA desktop cytometer (Guava, Hayward, CA), equal amounts of each analyte combined in multiplex tubes, and stored at 3,000 microspheres/μL at 4° C, protected from light.

#### Luminex assay and standard curves

The SAT assay developed for these analyses used modified standard curves and a template employed by the Laboratory of Malaria and Vector Research (LMVR, NIH) ELISA reference center [33]. Briefly, 3,000 microspheres per analyte were added to a 96-well round bottom plate per well. A HIP pool from Mozambican volunteers was applied in a 2-fold serial dilution for a starting dilution of 1∶1,500 and incubated for 1 h at room temperature with plate agitation and protection from light. The plate was washed by pelleting microspheres (centrifuge at 800×*g* for 5 min) and resuspended with wash buffer (0.05% Tween 20/PBS). 100 µL of biotinylated anti-human IgG (Sigma, Tres Cantos, Spain) diluted 1∶2,500 in assay buffer was applied to all wells and incubated at room temperature for 45 min with agitation and protection from light. The plate was washed as before, and 100 µL of streptavidin-conjugated R-phycoerythrin (Invitrogen, Carlsbad, CA) diluted 1∶1,000 in assay buffer was added and incubated at room temperature for 25 min with agitation and protection from light. The plate was immediately read using Bio-Plex Manager version 4.0, and at least 100 microspheres per analyte were acquired per sample. Crude mean fluorescent intensity (MFI) was exported with background fluorescence from blank wells already subtracted. Additionally, Bio-Plex Manager software automatically calculated the regression equation for the curves of each analyte. The 5-parameter logistic regression curve with logarithmic variance weighting was selected due to superior fit with antibody data [34], [35].
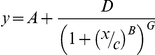
where A is the lower asymptote, B is the slope at the inflection point, C is the concentration at the inflection point, D is the upper asymptote, and G is a factor of asymmetry.

The back-calculated dilution corresponding to 15,000 MFI, roughly the middle point of the linear region of a fully saturated sigmoidal curve with the Bio-Plex 100 system, was assigned the arbitrary value of 15,000 Antibody Units (AU), and the "undiluted units" of the HIP pool for each analyte was calculated by multiplying 15,000 units by the corresponding dilution factor. This value was registered with the HIP pool to create a reference standard for these antigens. This reference standard was repeated on each assay day in a 14-point 2-fold dilution series, starting at a dilution of 1∶1,500 with the corresponding unit values (the large range of the dilution series encompasses different saturation points for each antigen). Test plasmas were incubated with the multiplex microspheres at a final dilution of 1∶500 in duplicate, and the assay was performed as described for the standard curves. Based on the standard curves for each analyte, AU concentration for each antigen was calculated by the Bio-Plex Manager software. Samples where microsphere aggregation exceeded 50% were excluded from analysis.

### Anti-VSA antibody assays

VSA antibody assays were performed as described previously [36]. The R29 line of *P. falciparum* was selected for anti-VSA assays because, compared to other field and laboratory isolates, it was the most immunogenic in preliminary studies in this population (Dobaño et al. unpublished). Briefly, synchronized cultures of the R29 line were cryopreserved and thawed for each assay day. Sterile 96-well round bottom plates were blocked with 200 µL/well of 1% BSA/PBS and left overnight at 4° C. Erythrocyte pellets were measured and resuspended in 1% BSA/PBS to 1% hematocrit, and 95 µL of erythrocyte suspension was added to each well. A 1∶20 dilution, or 5 µL of serum, was added to wells without replicates, and 4 wells were left as controls: a blank, two compensation controls and a HIP positive control. Plates were incubated for 30 min at room temperature with mild agitation and washed three times by centrifuging the plate at 1,200×*g* for 2 min, carefully flicking off the supernatant, and resuspending the pellets with 200 µL of 1% BSA/PBS. Pellets were resuspended in 100 µL of polyclonal rabbit anti-human IgG (Dako Cytomation, Glostrup, Denmark) diluted in 1∶200 in 1% BSA/PBS and incubated for 30 min at room temperature with gentle agitation. Plates were washed and pellets resuspended in 100 µL of Alexa Fluor 488 donkey anti-rabbit IgG (Invitrogen, Carlsbad, CA) diluted 1∶1,000 and ethidium bromide (Ecogen, Barcelona, Spain) diluted 1∶1,000 from a 1% stock in 1% BSA/PBS and incubated for 30 min at room temperature with gentle agitation and protection from light. Plates were washed as before and two additional times with PBS. Pellets were resuspended in 200 µL of PBS, transferred to cytometer acquisition tubes containing 200 µL of PBS, acquired on a 4-color FACS Calibur and analyzed using CellQuest Pro v5.2.1 (BD, Franklin Lakes, NJ). A gate for infected events was established using infected erythrocytes and ethidium bromide stain. A minimum of 1,000 infected events and up to 5,000 were acquired. Geometric mean MFI was reported for both infected and uninfected events, and an overall MFI value for specific VSA_R29_ antibodies was calculated by subtracting the MFI of uninfected events from MFI of infected events.

Procedures for normalization of data were as follows. A 6-step 2-fold serial dilution of HIP was added in duplicate to each assay plate, starting with a 1∶20 dilution to generate a reference curve for inter-assay variation and dynamic assay normalization. A standardized mean fluorescence intensity (MFI) score was assigned to each sample, as previously described [37], based on the hyperimmune plasma (HIP) titration. Samples with specific MFI above the highest titer (1∶20) were assigned a score of 6, between that and the next titer (1∶40) a score of 5, and so forth, and all samples with specific MFI below the lowest titer (1∶640) were given a score of 0. Additionally, the HIP titrations for all plates were averaged, and 6 "normalization constants" were generated, one for each dilution of the HIP, which were the ratio of MFI on the plate to the mean of all plates. All samples were normalized by multiplying the crude MFI by the normalization constant corresponding to the standardized MFI score for that sample (e.g. a sample with MFI score of 6 would be normalized with the normalization constant for the 1∶20 HIP dilution for that plate). This method allowed for more accurate adjustments across the MFI spectrum than by normalizing all values by a single dilution of a positive control.

### Statistical Analysis

All antibody data were exported in Microsoft Excel, organized and imported into STATA version 11 (STATA Corp., Texas). Luminex values were divided by 1,000 for analysis. Data was log-transformed using the natural logarithm. Differences between groups were analyzed by the reverse cumulative distribution function [38] and the Wilcoxon rank-sum test. Correlations between antibody responses to different antigens were assessed by Pearson's correlation coefficient (ρ), and p-values were adjusted by the Bonferroni correction. High correlation was considered for values of ρ greater than 0.7, and moderate correlation was considered for *ρ* greater than 0.5. Continuous data were analyzed for variables interacting with antibody levels using an Ordinary Least Square (OLS) linear regression model adjusting for age group, cohort, blood stage antibodies at baseline (based on IFAT performed for the main trial) [7], previous malaria episodes, present infection and batch of experiments. Interactions with the vaccine group and the adjusting variables were tested with an F-test. Data were categorized into 5 groups: below range, lower/middle/higher tertiles and above range. Categorical data were analyzed using an Ordered Politomous Logisitc Regression (OPLR) model adjusting for the same variables as above. Continuous and categorical data were compared for time-to-first clinical episode in the single-blind follow-up time period from 6–18 months post-vaccination [31] using univariate and step-wise multivariate Cox proportional hazard models on all children, stratified by cohort and adjusting for vaccine group, age group, IFAT at baseline, and previous clinical malaria episodes. Maternal antibodies were not considered as a confounding factor, because the youngest children in the study were nearly 2 years of age at the time of sampling. Results were considered statistically significant for a p-value <0.05.

## Results

Of the 2022 children aged 1-4 years enrolled, sera from 6 months post-vaccination in 580 randomized individuals who completed according-to-protocol (ATP) criteria throughout the single-blind phase (6–18 months post-vaccination) were assayed by Luminex and flow cytometry. Samples were evenly distributed between C1 and C2 and between the < 2 years and ≥2 years age groups.

### Antibody responses in RTS,S and control children

**Figure 1:**
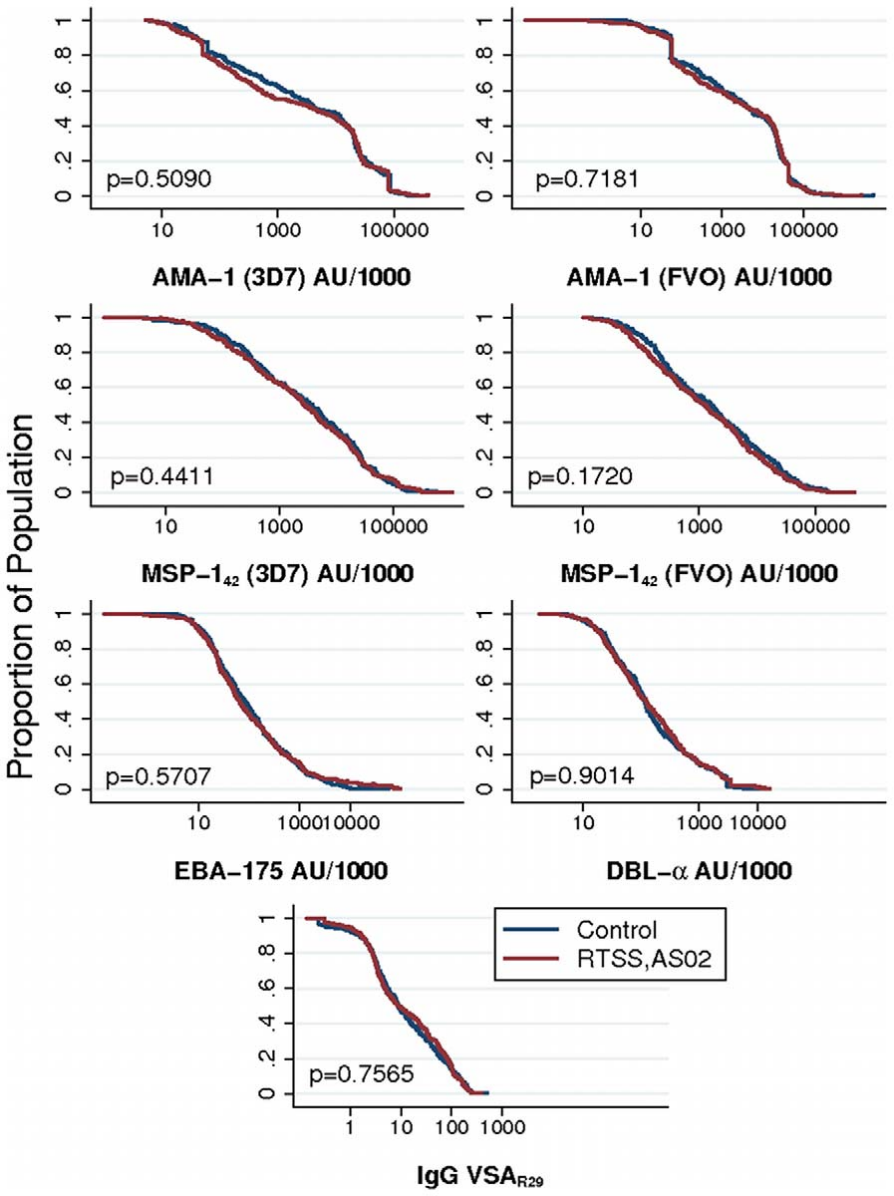
Reverse cumulative distribution of crude IgG responses by treatment group. The graphs represent the pooled antibody responses of children 1–4 years (cohorts 1 & 2 together) who received RTS,S/AS02 vaccine or control vaccine.

**Figure 2:**
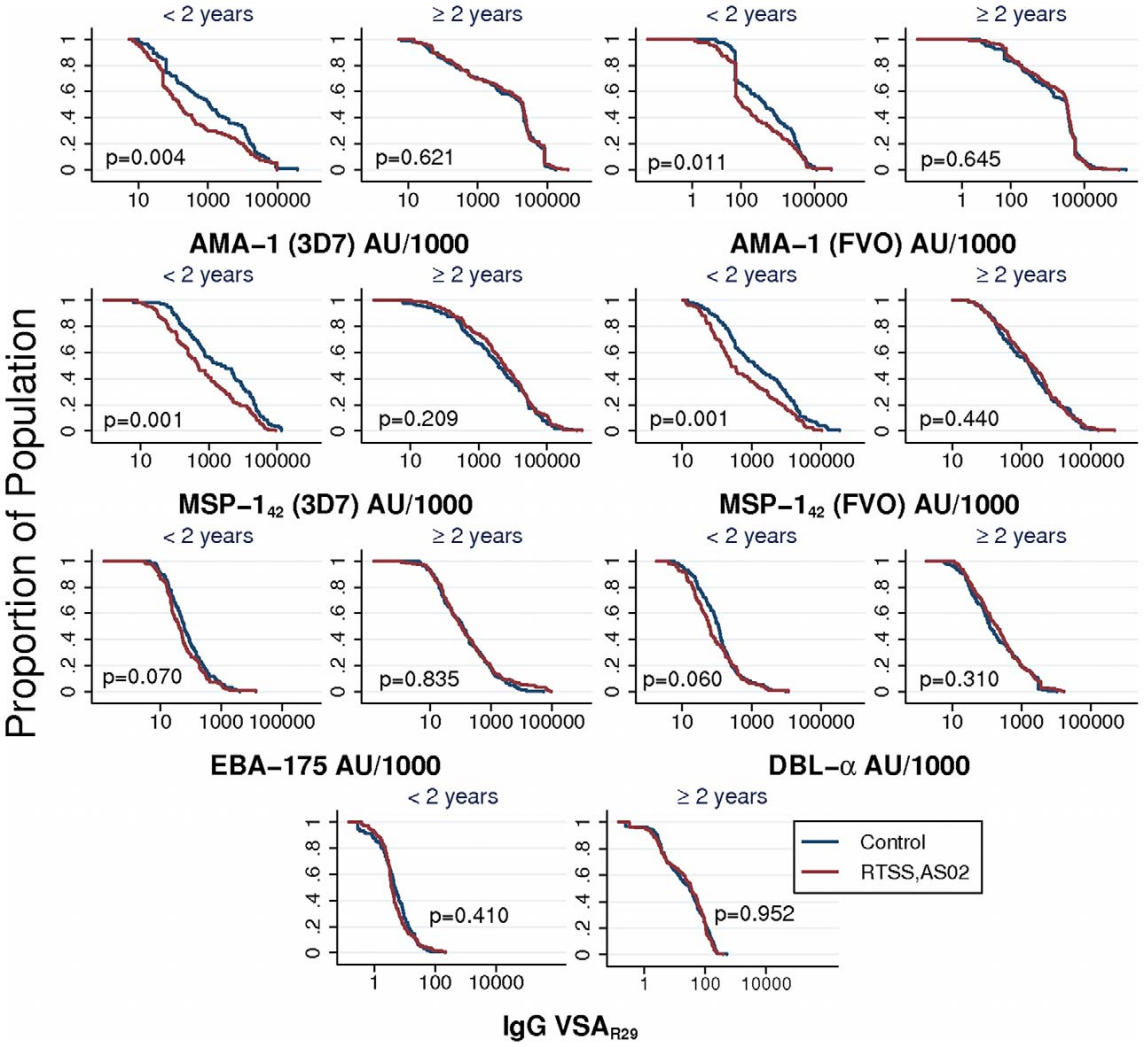
Reverse cumulative distribution of antibodies in RTS,S/AS02 vaccine vs. control vaccine groups, stratified by age group. The graphs represent the pooled antibody responses (cohorts 1 & 2) who received RTS,S/AS02 vaccine or control vaccine.

There were no significant differences in the distribution of IgG responses to any of the antigens tested between RTS,S/AS02 vaccine candidate and control groups in the crude (Figure 1 & Table S2), or adjusted (Table 1) analyses, and this was the case for both cohorts separately (Table S3). However, the effect of RTS,S/AS02 vaccination on blood stage antibodies was different by age group (Figure 2), with lower IgG levels for the 3D7 and FVO strains of AMA-1 (p = 0.0104 & p = 0.0147, respectively) and MSP-1_42_ (p = 0.0009 & p = 0.0142, respectively) in the RTS,S group compared to control, among younger (< 2 years) children but not in the older (≥ 2 years) children (Table 2 & Table S4). No difference was seen in either age group for antibodies against EBA-175, DBL-α, or VSA_R29_.

**Table 1:** Multivariate linear regression model showing the effect of RTS,S vaccination on levels of IgG to blood stage antigens, compared to control vaccination, adjusted by cohort, age, IFAT titer at baseline, batch of experiments, previous episodes and present infection.

Antigen	Prop. Diff^a^	95% CI^b^	p	R^2^ c
AMA-1 (3D7)	0.82	0.61–1.10	0.190	0.63
AMA-1 (FVO)	0.90	0.66–1.24	0.525	0.62
MSP-1_42_ (3D7)	0.97	0.72–1.32	0.867	0.48
MSP-1_42_ (FVO)	0.87	0.64–1.18	0.365	0.34
EBA-175	1.08	0.83–1.41	0.547	0.38
DBL-α	1.13	0.91–1.40	0.258	0.43
VSA_R29_	1.07	0.87–1.33	0.502	0.49

a Proportional difference refers to the proportional effect per log-increase in antibody level.

b Confidence Interval.

c R^2^ value of the OLS regression model was <0.65 in all cases, indicating that only a portion of the variability of IgG data is explained in the model.

**Table 2:** F-test for interactions between RTS,S vaccine group and key variables on antibody levels.

	Prop. Diff.^a^ (95% CI) <2 years	Prop. Diff.^a^ (95% CI) >2 years	p-value interaction RTS,S x Age group
AMA-1 (3D7)	0.51 (0.32–0.81)	1.11 (0.76–1.63)	0.0104
AMA-1 (FVO)	0.55 (0.34–0.91)	1.23 (0.82–1.84)	0.0147
MSP-1_42_ (3D7)	0.52 (0.33–0.82)	1.46 (0.98–2.17)	0.0009
MSP-1_42_ (FVO)	0.54 (0.34–0.87)	1.17 (0.79–1.74)	0.0142

Results are proportional difference in antibody levels associated with being in the RTS,S/AS02 vaccine group.

a Proportional difference refers to the proportional effect per log-increase in antibody level to blood stage antigens; CI: Confidence Interval.

As previously shown, antibodies were generally higher in the older age groups, in the region of higher transmission intensity (C2), in children with previous malaria episodes or infection at the time of sampling, and in those with higher pre-vaccination IFAT titers (Table S5).

### Correlation of antibody responses to different antigens

**Figure 3:**
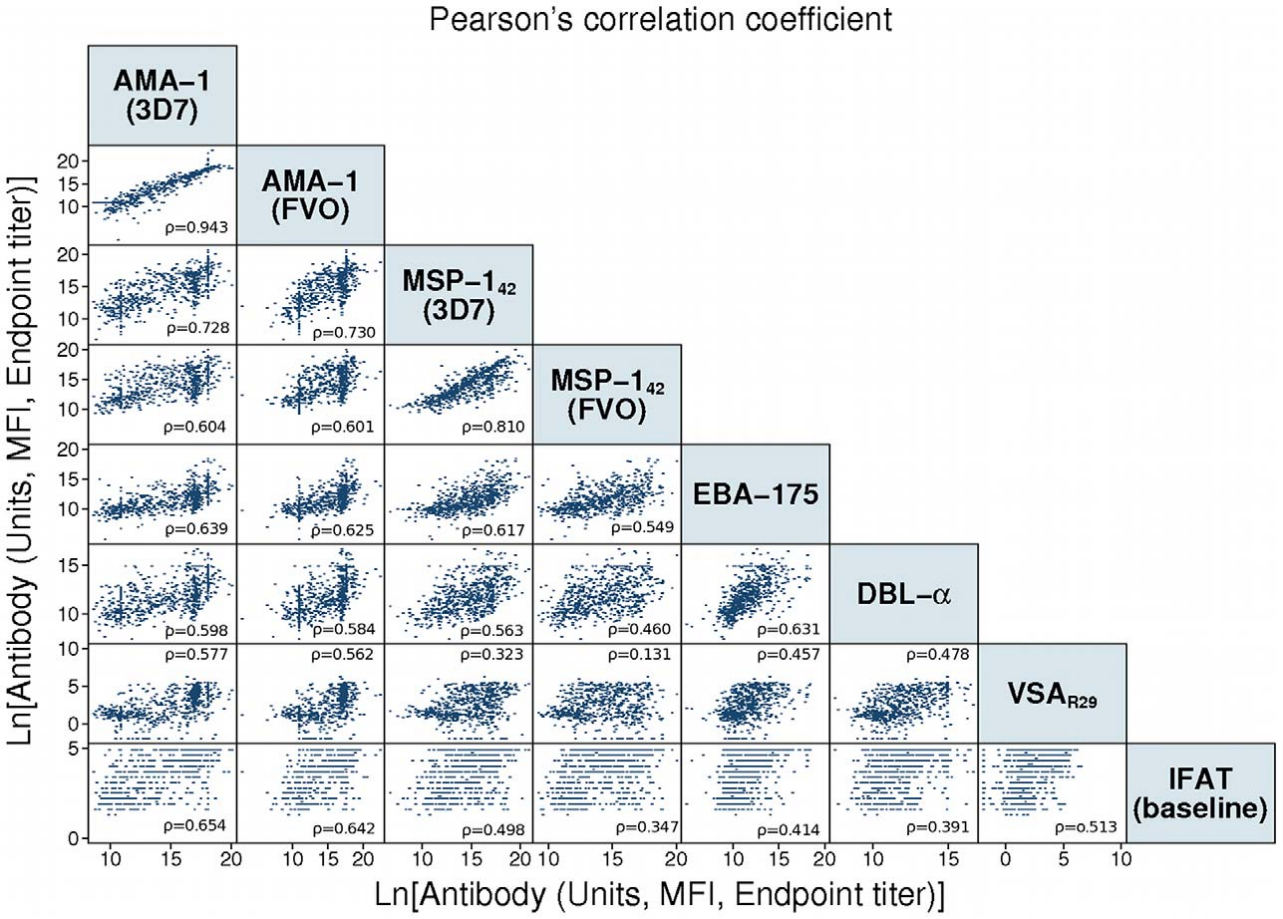
Correlation of antibody responses to merozoite and VSA antigens within the individual. A matrix of log-transformed antibody units or mean fluorescence intensities (VSA_R29_) against the selected blood stage antigens shows correlation of antibody responses between antigens. Pearson's correlation coefficient (*ρ*) is included in each antibody combination panel (p-value<0.001 for all correlations).

Antibody responses to the different antigens were not systematically correlated (Figure 3). Most antibody combinations were moderately (15/28) to highly (4/28) correlated. Correlation was high between AMA-1 3D7 and FVO (*ρ*: 0.94, p<0.001) or MSP-1_42_ 3D7 and FVO strains (*ρ*: 0.81, p<0.001), indicating high levels of cross-strain recognition. Prior to performing assays, HIP was titrated against AMA-1 and MSP-1_42_ 3D7- and FVO-coated microspheres in singleplex and multiplex to confirm a minimal level of cross-reactivity in the positive control plasma (data not shown).

### Antibody responses in relation to protection from clinical malaria

We calculated the effect of antibodies at 6 months post-vaccination on the hazard of clinical malaria, analyzed as continuous and categorized data. There were no divergences in the continuous or categorical models, and only the continuous models are reported here. In C1, there was no evidence of any association between antibodies and risk of clinical malaria. The hazard to the first or only episode of clinical malaria was significantly higher in children with previous malaria episodes (hazard ratio [HR]: 2.95, p<0.001), and lower in the older age group of children (HR: 0.54, p = 0.015). In C2, there was a lower risk of clinical malaria associated with higher IgG to EBA-175 and VSA_R29_ (Table 3 & Table S6), as well as a lower risk in the older age group (HR: 0.57, p = 0.041). The HR was, again, significantly higher in children with previous malaria episodes (HR: 1.91, p = 0.004).

**Table 3:** Cox proportional hazards model showing effect of IgG levels on risk of having a clinical malaria episode from 6–18 months post-vaccination.

	HR^a^	p	95% CI^b^
Cohort 1			
AMA-1 (3D7)	0.97	0.409	0.90–1.05
AMA-1 (FVO)	0.99	0.731	0.92–1.06
MSP-1_42_ (3D7)	0.95	0.160	0.87–1.02
MSP-1_42_ (FVO)	0.94	0.107	0.87–1.01
EBA-175	1.01	0.910	0.92–1.10
DBL-α	0.97	0.649	0.87–1.09
VSA_R29_	1.12	0.055	1.00–1.26
Cohort 2			
AMA-1 (3D7)	0.97	0.577	0.88–1.07
AMA-1 (FVO)	0.97	0.589	0.88–1.07
MSP-1_42_ (3D7)	0.92	0.033	0.85–0.99
MSP-1_42_ (FVO)	0.92	0.056	0.85–1.00
EBA-175^b^	0.81	0.000	0.74–0.90
DBL-α	0.92	0.099	0.83–1.02
VSA_R29_ b	0.80	0.001	0.70–0.92

Only the univariate model is shown.

a Hazard Ratio is the proportional effect on the hazard per doubling of antibody levels.

b Confidence Interval. ^c^Significant in step-wise multivariate model (adjusted by vaccination group, age, previous clinical malaria cases and baseline immunofluorescence antibody test titers); VSA = Variant surface antigen.

## Discussion

We tested a hypothesis that vaccination of children with the partially protective, pre-erythrocytic stage RTS, S adjuvanted vaccine candidate would elicit a broadly stronger asexual blood stage immune response that may be associated with long-term protection from clinical disease. Furthermore, we proposed that this observation would explain the waning efficacy observed in C2 and in newborns [8], [12]. From the onset, we were confronted with the question of which antigens to select among the nearly 2,000 antigens of the asexual blood stage (www.plasmodb.org). Our selection was limited by the current knowledge and availability of blood stage antigens, and we used targets expressed on the surface of merozoites or infected erythrocytes, thus accessible to the immune system and probably under immune pressure [39], and which have known functions in erythrocyte invasion, immune evasion and cytoadherence.

No differences were observed in the distribution of antibodies to AMA-1 (3D7 & FVO), MSP-1_42_ (3D7 & FVO), EBA-175, DBL-α and VSA_R29_ when comparing all children in the RTS,S/AS02 and control vaccine groups, suggesting that RTS,S/AS02 neither enhances nor impairs antibodies naturally acquired against blood stage antigens. This finding contrasts with a recent report on blood stage antibodies in a Phase IIb trial of RTS,S in Kenya and Tanzania, which found a significant reduction in blood stage antibodies in RTS,S-immunized children [40]. However, when stratifying by age, AMA-1 and MSP-1_42_ antibodies were lower in the RTS,S/AS02 group in children <2 years of age at enrollment. In this case, the results from Bejon *et al*. agree with ours, given that the ages of all children in that trial were below 2 years of age [9]. The reduction in episodes of malaria and, thus, exposure to parasites afforded by RTS, S/AS02, thereby reduces levels of IgG to some blood stage antigens, particularly antigens shown to be markers of exposure [41]–[43]. The absence of a detectable reduction in the older age group may be explained by the relatively high antibody levels already acquired in these children; reduced exposure in the RTS, S group may have a less dramatic effect on these antibody levels.

The hypothesis that waning efficacy in C2 is a result of interrupted exposure to low dose parasitemia hinges on a differential profile of blood stage antibodies from those of C1, which is not supported by these data. One explanation is that these antigens may merely serve as strong markers of exposure, which is expected to be lower overall in the RTS,S group. IgG to all antigens tested were higher in C2 and may indicate an already advanced level of blood stage immunity, where RTS,S gives less impact on blood stage antibodies. Lower antibodies in younger children may also be linked to exposure. Whereas blood stage antibodies to AMA-1 and MSP-1_42_ in older children may have already reached a plateau, partial pre-erythrocytic protection afforded by RTS,S may reduce the levels of antibodies to antigens that are strongly associated with exposure in the younger children. We have no evidence that these lower levels of antibodies in younger RTS,S-vaccinated children result in an impairment of blood stage immunity over the 43 months of follow-up. Further studies should confirm this result. The difference in transmission intensity between cohorts remains a potential factor in vaccine efficacy that cannot be controlled in these data, but which may explain the waning efficacy observed in C2 through potential mechanisms such as increased likelihood of inoculation, greater exposure to parasite genetic diversity and a higher level of challenge to strain-specific or variant-specific naturally acquired immunity.

Interestingly, children with higher IgG to EBA-175 and VSA_R29_ at 6 months post-vaccination had a lower hazard of clinical malaria up to 18 months, but this result was only observed in C2. We found that vaccine group did not modify antibody levels to these antigens differently in C1 and C2, thus showing no interaction between vaccine group and cohort and suggesting that protection in C1 may be mediated by other immune factors. It is possible that the children living in Ilha Josina (C2) experience a faster build-up of immunity than those living in Manhiça (C1) due to the differences in transmission intensity, perhaps indicating a more “mature” immune response in C2. This is supported by the clinical patterns of disease in these populations [44], where the age pattern of disease is shifted to earlier age in Ilha Josina, compared to Manhiça (Aide *et al*., Guinovart *et al*., in preparation). It follows that affinity maturation of antibodies in C2 may also occur earlier than in C1 and account for differences in the protective effect of the antibodies. Thus, further characterization of the antibodies evaluated here, particularly fine specificity [45], subclasses of IgG [29], affinity and/or avidity [46] and in vitro functional capacity, could show further differences, both between cohorts and in prospective risk of disease.

Could other asexual blood stage antigens be more relevant to immunity? Evidence from a study in Mali before and after the transmission season suggests that a number of previously uncharacterized antigens may be better candidates for discrimination of protection from clinical disease [47]. A broader repertoire of antibody responses is shown to be associated with reduced risk of clinical malaria [48]. Anti-VSA antibodies and, particularly, how rapidly they are acquired in succession, may be important factors in parasite control [49]. The moderate correlation between antibodies against different antigens suggests that the selection may not be representative of the blood stage antibody immune response as a whole, and that different antibody levels could be expected within individuals for other antigens. It remains to be seen if these antibodies are acquired differently in RTS,S-vaccinated versus control-vaccinated individuals.

In conclusion, antibodies to the immunodominant blood stage antigens evaluated here were neither higher nor lower in RTS,S-immunized children compared to the control vaccine group, as a whole. In younger children, there seemed to be a reduction in antibodies against some antigens in the RTS,S group, although there is no evidence in this study that these lower antibody levels translate to an impairment of naturally acquired immunity. Based on our findings, antibodies to the common blood stage antigens tested do not explain the difference in long-term efficacy between C1 and C2. The data are not sufficient to reject the hypothesis that the RTS,S vaccine facilitates immunity upon exposure to blood stage parasites, as it is not known if the antigens selected are representative of, or relevant to, the overall protective antibody response of asexual blood stage antigens, and CMI responses were not addressed. Further characterization of naturally acquired blood stage antibodies must be done for the duration of the follow-up period to highlight protective antibody responses and effects of RTS,S vaccination in these two cohorts.

## Supporting Information

Table S1 **Baseline characteristics of sample selection.**
(DOCX)

Table S2 **Univariate analysis of vaccine group and antibody levels are presented in Tables S2A-S2G.** Data are presented as geometric mean antibody units or geometric mean of mean fluorescence intensity with standard deviations (SD).
(DOCX)

Table S3 **Univariate analysis of vaccine group and antibody levels by study cohort.**
(DOCX)

Table S4 F-test for interaction between RTS,S vaccine group and adjusting variables of multivariate linear regression model. Data are displayed as p-values, where p<0.05 is considered a significant interaction.
(DOCX)

Table S5 **Multivariate analysis of vaccine group and antibody levels.** The linear regression model was adjusted by cohort, age, IFAT titer at baseline, batch of experiments, previous episodes and present infection.
(DOCX)

Table S6 **Cox proportional hazards model showing effect of IgG levels on risk of having a clinical malaria episode from 6 – 18 months post-vaccination using a multivariate step-wise model adjusting for treatment, cohort, age group, and previous clinical malaria episodes.**
(DOCX)
