# Supplementary material for: Impact of the RTS,S Malaria Vaccine Candidate on Naturally Acquired Antibody Responses to Multiple Asexual Blood Stage Antigens
Source: PLoS One. 2011 Oct 12;6(10):e25779. doi: 10.1371/journal.pone.0025779 (PMC3192128; doi:10.1371/journal.pone.0025779)
Supplement: Table S1 — Baseline characteristics of sample selection. (DOCX) [file pone.0025779.s001.docx]

**Table S1.** Baseline characteristics of sample selection.

|  |  | Control vaccine | RTS,S vaccine | p-value |
| --- | --- | --- | --- | --- |
| Age group | < 2 year | 117 (42%) | 110 (36%) | 0.163 |
|  | 2-4 year | 161 (58%) | 192 (64%) |  |
| Previous clinical malaria | 0 | 189 (68%) | 224 (74%) | 0.102 |
|  | ≥ 1 | 89 (32%) | 78 (26%) |  |
| Previous malaria infection | 0 | 111 (40%) | 130 (43%) | 0.415 |
|  | ≥ 1 | 167 (60%) | 172 (57%) |  |
| Infection M8.5^a^ | Neg | 184 (66%) | 217 (72%) | 0.140 |
|  | Pos | 94 (34%) | 85 (28%) |  |
| Baseline^b^ IFAT titer^c^ |  | 4469 (11509) | 5329 (13298) | 0.405 |

^a^Study month 8.5 corresponding to 6 months post-vaccination. ^b^Baseline sampling corresponding to sampling before first dose of RTS,S or control vaccine. ^c^Result displayed as endpoint titer and standard deviation in parenthesis
