# Supplementary material for: Impact of the RTS,S Malaria Vaccine Candidate on Naturally Acquired Antibody Responses to Multiple Asexual Blood Stage Antigens
Source: PLoS One. 2011 Oct 12;6(10):e25779. doi: 10.1371/journal.pone.0025779 (PMC3192128; doi:10.1371/journal.pone.0025779)
Supplement: Table S2 — Univariate analysis of vaccine group and antibody levels are presented in Tables S2A-S2G. Data are presented as geometric mean antibody units or geometric mean of mean fluorescence intensity with standard deviations (SD). (DOCX) [file pone.0025779.s002.docx]

**Table S2.** Univariate analysis of vaccine group and antibody levels are presented in Tables S2A-S2G. Data are presented as geometric mean antibody units or geometric mean of mean fluorescence intensity with standard deviations (SD).

| **Table S2A.** AMA-1 (3D7). | |  |  |
| --- | --- | --- | --- |
|  | Control Vaccine Geometric Mean (SD) | RTS,S Vaccine Geometric Mean (SD) | p-value |
| AMA-1 (3D7) | 2424.7 (6913.2) | 1893.4 (5680.2) | 0.509 |
| <2 year | 1057.4 (2947.4) | 349.6 (962.8) | 0.004 |
| ≥ 2 year | 4431.7 (12194.2) | 4984.4 (13437.7) | 0.621 |
| C1 | 730.7 (2028.7) | 462.8 (1312.9) | 0.131 |
| C2 | 19843.7 (27633.5) | 17035.6 (27340.8) | 0.784 |
| **Table S2B.** AMA-1 (FVO). | |  |  |
|  | Control Vaccine Geometric Mean (SD) | RTS,S Vaccine Geometric Mean (SD) | p-value |
| AMA-1 (FVO) | 2231.7 (6646.6) | 1883.5 (5970.9) | 0.718 |
| <2 year | 971.8 (2689.8) | 334.9 (1023.8) | 0.011 |
| ≥ 2 year | 4083.5 (12202.0) | 5066.5 (14150.8) | 0.645 |
| C1 | 631.7 (1819.9) | 463.3 (1395.0) | 0.299 |
| C2 | 20382.6 (30229.0) | 16780.3 (32509.0) | 0.752 |
| **Table S2C.** MSP-1_42_ (3D7). | |  |  |
|  | Control Vaccine Geometric Mean (SD) | RTS,S Vaccine Geometric Mean (SD) | p-value |
| MSP-1_42_ (3D7) | 2618.6 (6629.5) | 2281.5 (5904.8) | 0.441 |
| <2 year | 2077.9 (4999.3) | 666.9 (1628.1) | 0.001 |
| ≥ 2 year | 3097.9 (8097.4) | 4615.6 (11101.8) | 0.209 |
| C1 | 1337.4 (3486.7) | 829.5 (2036.5) | 0.058 |
| C2 | 8500.9 (16111.5) | 11049.3 (21217.8) | 0.344 |
| **Table S2D.** MSP-1_42_ (FVO). | |  |  |
|  | Control Vaccine Geometric Mean (SD) | RTS,S Vaccine Geometric Mean (SD) | p-value |
| MSP-1_42_ (FVO) | 1581.5 (3575.3) | 1213.9 (2785.1) | 0.172 |
| <2 year | 1570.3 (3838.9) | 560.9 (1336.2) | 0.001 |
| ≥ 2 year | 1589.7 (3378.0) | 1889.1 (4015.2) | 0.440 |
| C1 | 1021.8 (2433.6) | 639.5 (1504.3) | 0.050 |
| C2 | 3400.5 (6124.2) | 3297.2 (5931.1) | 0.948 |
| **Table S2E.** EBA-175. | |  |  |
|  | Control Vaccine Geometric Mean (SD) | RTS,S Vaccine Geometric Mean (SD) | p-value |
| EBA-175 | 101.6 (189.6) | 100.3 (214.7) | 0.571 |
| <2 year | 72.2 (109.0) | 50.3 (80.3) | 0.070 |
| ≥ 2 year | 130.2 (267.7) | 148.9 (344.0) | 0.835 |
| C1 | 55.6 (92.1) | 56.5 (119.2) | 0.556 |
| C2 | 292.4 (509.8) | 245.4 (459.2) | 0.437 |
| **Table S2F.** DBL-α. | |  |  |
|  | Control Vaccine Geometric Mean (SD) | RTS,S Vaccine Geometric Mean (SD) | p-value |
| DBL-α | 138.5 (230.5) | 144.6 (251.5) | 0.901 |
| <2 year | 104.7 (149.2) | 75.1 (119.7) | 0.060 |
| ≥ 2 year | 169.7 (304.8) | 210.4 (360.2) | 0.310 |
| C1 | 78.3 (111.9) | 84.8 (137.8) | 0.975 |
| C2 | 376.3 (594.5) | 332.0 (526.8) | 0.392 |
| **Table S2G.** VSA_R29_. | |  |  |
|  | Control Vaccine Geometric Mean (SD) | RTS,S Vaccine Geometric Mean (SD) | p-value |
| VSA_R29_ | 10.9 (19.7) | 12.0 (21.4) | 0.757 |
| <2 year | 4.5 (6.2) | 4.5 (5.4) | 0.410 |
| ≥ 2 year | 20.7 (37.4) | 21.0 (38.4) | 0.952 |
| C1 | 4.9 (7.8) | 5.9 (9.3) | 0.457 |
| C2 | 44.6 (52.9) | 36.3 (55.3) | 0.736 |
