# Supplementary material for: Impact of the RTS,S Malaria Vaccine Candidate on Naturally Acquired Antibody Responses to Multiple Asexual Blood Stage Antigens
Source: PLoS One. 2011 Oct 12;6(10):e25779. doi: 10.1371/journal.pone.0025779 (PMC3192128; doi:10.1371/journal.pone.0025779)
Supplement: Table S3 — Univariate analysis of vaccine group and antibody levels by study cohort. (DOCX) [file pone.0025779.s003.docx]

**Table S3.** Univariate analysis of vaccine group and antibody levels by study cohort.

|  | Cohort | Control Vaccine Geometric Mean (SD)^a^ | RTS,S Vaccine Geometric Mean (SD) | p-value^b^ |
| --- | --- | --- | --- | --- |
| AMA-1 (3D7) | C1 | 730.7 (2028.7) | 462.8 (1312.9) | 0.131 |
|  | C2 | 19843.7 (27633.5) | 17035.6 (27340.8) | 0.784 |
| AMA-1 (FVO) | C1 | 631.7 (1819.9) | 463.3 (1395.0) | 0.299 |
|  | C2 | 20382.6 (30229.0) | 16780.3 (32509.0) | 0.752 |
| MSP-1_42_ (3D7) | C1 | 1337.4 (3486.7) | 829.5 (2036.5) | 0.058 |
|  | C2 | 8500.9 (16111.5) | 11049.3 (21217.8) | 0.344 |
| MSP-1_42_ (FVO) | C1 | 1021.8 (2433.6) | 639.5 (1504.3) | 0.050 |
|  | C2 | 3400.5 (6124.2) | 3297.2 (5931.1) | 0.948 |
| EBA-175 | C1 | 55.6 (92.1) | 56.5 (119.2) | 0.556 |
|  | C2 | 292.4 (509.8) | 245.4 (459.2) | 0.437 |
| DBL-α | C1 | 78.3 (111.9) | 84.8 (137.8) | 0.975 |
|  | C2 | 376.3 (594.5) | 332.0 (526.8) | 0.392 |
| VSA_R29_ | C1 | 4.9 (7.8) | 5.9 (9.3) | 0.457 |
|  | C2 | 44.6 (52.9) | 36.3 (55.3) | 0.736 |

^a^SD: standard deviation. ^b^Wilcoxon rank-sum test
