# Supplementary material for: Impact of the RTS,S Malaria Vaccine Candidate on Naturally Acquired Antibody Responses to Multiple Asexual Blood Stage Antigens
Source: PLoS One. 2011 Oct 12;6(10):e25779. doi: 10.1371/journal.pone.0025779 (PMC3192128; doi:10.1371/journal.pone.0025779)
Supplement: Table S4 — F-test for interaction between RTS,S vaccine group and adjusting variables of multivariate linear regression model. Data are displayed as p-values, where p<0.05 is considered a significant interaction. (DOCX) [file pone.0025779.s004.docx]

**Table S4.** F-test for interaction between RTS,S vaccine group and adjusting variables of multivariate linear regression model. Data are displayed as p-values, where p < 0.05 is considered a significant interaction.

|  | RTS,S x Cohort | RTS,S x Age group | RTS,S x IFAT | RTS,S x Batch |
| --- | --- | --- | --- | --- |
| AMA-1 (3D7) | 0.1681 | 0.0104 | 0.7309 | 0.4151 |
| AMA-1 (FVO) | 0.5264 | 0.0147 | 0.6313 | 0.4705 |
| MSP-1_42_ (3D7) | 0.0235 | 0.0009 | 0.6364 | 0.9107 |
| MSP-1_42_ (FVO) | 0.2047 | 0.0142 | 0.9922 | 0.4531 |
| EBA-175 | 0.4738 | 0.6170 | 0.9361 | 0.6994 |
| DLB-α | 0.3574 | 0.2883 | 0.8707 | 0.3095 |
| R29 | 0.2758 | 0.6977 | 0.8118 | 0.8771 |
