# Supplementary material for: Impact of the RTS,S Malaria Vaccine Candidate on Naturally Acquired Antibody Responses to Multiple Asexual Blood Stage Antigens
Source: PLoS One. 2011 Oct 12;6(10):e25779. doi: 10.1371/journal.pone.0025779 (PMC3192128; doi:10.1371/journal.pone.0025779)
Supplement: Table S5 — Multivariate analysis of vaccine group and antibody levels. The linear regression model was adjusted by cohort, age, IFAT titer at baseline, batch of experiments, previous episodes and present infection. (DOCX) [file pone.0025779.s005.docx]

**Table S5.** Multivariate analysis of vaccine group and antibody levels. The linear regression model was adjusted by cohort, age, IFAT titer at baseline, batch of experiments, previous episodes and present infection.

| Antigen | Prop. Diff^a^ | 95% CI^b^ | p | R^2 c^ | Adjusting variables^d^ | Prop. Diff | 95% CI | p |
| --- | --- | --- | --- | --- | --- | --- | --- | --- |
| AMA-1 (3D7) | 0.82 | 0.61 – 1.10 | 0.190 | 0.63 | Cohort 2 | 2.09 | 1.36 – 3.23 | 0.001 |
|  |  |  |  |  | Age (>2 y) | 1.84 | 1.27 – 2.65 | 0.001 |
|  |  |  |  |  | IFAT-M0 | 2.51 | 2.06 – 3.06 | 0.000 |
|  |  |  |  |  | Prev Inf | 4.79 | 3.10 – 7.39 | 0.000 |
|  |  |  |  |  | Curr Inf | 3.42 | 2.27 – 5.16 | 0.000 |
| AMA-1 (FVO) | 0.90 | 0.66 – 1.24 | 0.525 | 0.62 | Cohort 2 | 1.83 | 1.13 – 2.96 | 0.014 |
|  |  |  |  |  | Age (>2 y) | 1.86 | 1.25 – 2.77 | 0.002 |
|  |  |  |  |  | IFAT-M0 | 2.53 | 2.06 – 3.10 | 0.000 |
|  |  |  |  |  | Prev Inf | 6.48 | 4.17 – 10.06 | 0.000 |
|  |  |  |  |  | Curr Inf | 3.59 | 2.31 – 5.60 | 0.000 |
| MSP-1_42_ (3D7) | 0.97 | 0.72 – 1.32 | 0.867 | 0.48 | Cohort 2 | 1.08 | 0.71 – 1.64 | 0.728 |
|  |  |  |  |  | Age (>2 y) | 1.30 | 0.91 – 1.87 | 0.146 |
|  |  |  |  |  | IFAT-M0 | 1.79 | 1.47 – 2.16 | 0.000 |
|  |  |  |  |  | Prev Inf | 6.02 | 3.97 – 9.13 | 0.000 |
|  |  |  |  |  | Curr Inf | 2.30 | 1.54 – 3.43 | 0.000 |
| MSP-1_42_ (FVO) | 0.87 | 0.64 – 1.18 | 0.365 | 0.34 | Cohort 2 | 0.85 | 0.53 – 1.36 | 0.497 |
|  |  |  |  |  | Age (>2 y) | 1.01 | 0.72 – 1.43 | 0.935 |
|  |  |  |  |  | IFAT-M0 | 1.42 | 1.16 – 1.72 | 0.001 |
|  |  |  |  |  | Prev Inf | 4.53 | 2.90 – 7.09 | 0.000 |
|  |  |  |  |  | Curr Inf | 2.07 | 1.39 – 3.07 | 0.000 |
| EBA-175 | 1.08 | 0.83 – 1.41 | 0.547 | 0.38 | Cohort 2 | 1.11 | 0.74 – 1.67 | 0.620 |
|  |  |  |  |  | Age (>2 y) | 1.36 | 1.06 – 1.76 | 0.018 |
|  |  |  |  |  | IFAT-M0 | 1.37 | 1.17 – 1.59 | 0.000 |
|  |  |  |  |  | Prev Inf | 2.82 | 2.01 – 3.94 | 0.000 |
|  |  |  |  |  | Curr Inf | 2.27 | 1.58 – 3.24 | 0.000 |
| DBL-α | 1.13 | 0.91 – 1.40 | 0.258 | 0.43 | Cohort 2 | 1.46 | 1.06 – 2.03 | 0.022 |
|  |  |  |  |  | Age (>2 y) | 1.41 | 1.12 – 1.78 | 0.004 |
|  |  |  |  |  | IFAT-M0 | 1.21 | 1.07 – 1.37 | 0.003 |
|  |  |  |  |  | Prev Inf | 1.89 | 1.47 – 2.43 | 0.000 |
|  |  |  |  |  | Curr Inf | 2.17 | 1.59 – 2.96 | 0.000 |
| VSA_R29_ | 1.07 | 0.87 – 1.33 | 0.502 | 0.49 | Cohort 2 | 2.21 | 1.59 – 3.05 | 0.000 |
|  |  |  |  |  | Age (>2 y) | 2.04 | 1.58 – 2.65 | 0.000 |
|  |  |  |  |  | IFAT-M0 | 1.47 | 1.27 – 1.69 | 0.000 |
|  |  |  |  |  | Prev Inf | 1.25 | 0.91 – 1.71 | 0.171 |
|  |  |  |  |  | Curr Inf | 2.78 | 2.07 – 3.72 | 0.000 |

^a^Proportional difference refers to the proportional effect per log-increase in antibody level. ^b^Confidence Interval. ^c^R^2^ value of the OLS regression model was < 0.65 in all cases, indicating that only a portion of the variability of IgG data is explained in the model. ^d^IFAT-M0 = immunofluorescence antibody test against blood stage parasites at baseline, Prev Inf = previous infection, Curr Inf = current infection.
