# Supplementary material for: Impact of the RTS,S Malaria Vaccine Candidate on Naturally Acquired Antibody Responses to Multiple Asexual Blood Stage Antigens
Source: PLoS One. 2011 Oct 12;6(10):e25779. doi: 10.1371/journal.pone.0025779 (PMC3192128; doi:10.1371/journal.pone.0025779)
Supplement: Table S6 — Cox proportional hazards model showing effect of IgG levels on risk of having a clinical malaria episode from 6 – 18 months post-vaccination using a multivariate step-wise model adjusting for treatment, cohort, age group, and previous clinical malaria episodes. (DOCX) [file pone.0025779.s006.docx]

**Table S6.** Cox proportional hazards model showing effect of IgG levels on risk of having a clinical malaria episode from 6 – 18 months post-vaccination using a multivariate step-wise model adjusting for treatment, cohort, age group, and previous clinical malaria episodes.

|  |  | HR^a^ | p-value | 95% CI^b^ |
| --- | --- | --- | --- | --- |
| Cohort 1^c^ | |  |  |  |
|  | RTS,S group | 1.52 | 0.081 | 0.95 – 2.46 |
|  | ≥ 2 year | 0.54 | 0.015 | 0.33 – 0.89 |
|  | Previous malaria | 2.95 | < 0.001 | 1.79 – 4.87 |
|  | Baseline IFAT | 1.48 | 0.001 | 1.17 – 1.87 |
| Cohort 2^d^ | |  |  |  |
|  | RTS,S group | 0.92 | 0.731 | 0.59 – 1.45 |
|  | ≥ 2 year | 0.57 | 0.041 | 0.34 – 0.98 |
|  | Previous malaria | 1.91 | 0.004 | 1.23 – 2.96 |
|  | Baseline IFAT | 0.93 | 0.697 | 0.65 – 1.34 |
|  | EBA-175 | 0.76 | < 0.001 | 0.66 – 0.88 |
|  | VSA_R29_ | 0.75 | 0.005 | 0.62 – 0.92 |

^a^Hazard Ratio is the proportional effect on the hazard per doubling of antibody levels. ^b^Confidence Interval. ^c^Variables removed during the step-wise procedure: MSP-1_42_ 3D7 (p=0.9078), MSP-1_42_ FVO (0.4902), EBA-175 (p=0.5159), DBL-α (p=0.5191), AMA-1 FVO (p=0.5119), AMA-1 3D7 (p=0.1131), VSA_R29_ (p=0.0551). ^d^Variables removed during the step-wise procedure: AMA-1 FVO (p=0.7961), MSP-1_42_ FVO (p=0.6558), MSP-1_42_ 3D7 (p=0.3211), AMA-1 3D7 (p=0.3216), DBL-α (p=0.0986).
